# DTF-YOLO: A Thermal-Texture Decoupling and Recalibration Detector for Infrared-Visible Small Object Detection

**DOI:** 10.3390/s26216756

**Published:** 2026-10-26

**Authors:** Donglin He, Jingjing Zheng, Guangqun Zeng, Fengli Zhang, Ruijin Wang, Hui Li

**Affiliations:** 1 School of Aeronautics and Astronautics, University of Electronic Science and Technology of China, Chengdu 611731, China; hedonglin@caacsri.com (D.H.); kelly.li@126.com (H.L.); 2 The Second Research Institute of Civil Aviation Administration of China, Chengdu 610041, China; 3 School of Information and Software Engineering, University of Electronic Science and Technology of China, Chengdu 610051, China202322090402@std.uestc.edu.cn (G.Z.); ruijinwang@uestc.edu.cn (R.W.)

**Keywords:** object detection, multi-modal images, thermal-texture decoupling, small object detection, infrared–visible fusion, airport airside scenarios

## Abstract

All-weather, high-precision perception of airport airside targets such as distant personnel, ground vehicles, and small hazards is essential for safety control. Visible-light monitoring degrades under low illumination, dust, and fog, whereas infrared sensing captures heat-emitting targets but lacks scene texture; infrared–visible fusion has therefore become a mainstream route to robust detection. In fused images, however, thermal saliency and visible texture remain deeply entangled, modality interference varies across scenes and feature levels, and strided downsampling causes irreversible loss of small-target information. This paper proposes DTF-YOLO, a thermal-texture decoupling and recalibration detector built on a restructured YOLOv11 backbone. A Thermal-Texture Decoupling Recalibration (TTDR) module uses parallel thermal-radiation and texture-detail perception paths to recalibrate fused features at the channel and spatial level; a fully information-preserving Bi-directional Focus module replaces conventional downsampling with spatial-channel slice rearrangement; and a four-scale P2–P5 fusion neck improves sensitivity to tiny targets. Evaluated on the public M3FD and LLVIP datasets as proxy benchmarks, DTF-YOLO achieves 87.32% mAP@0.5 and 80.33% recall with 2.91 M parameters on M3FD, and 95.26% mAP@0.5 on LLVIP, demonstrating a favorable accuracy–efficiency trade-off and potential for airport airside safety monitoring.

## 1. Introduction

All-weather, high-precision perception of airside personnel, ground vehicles, aircraft, and foreign object debris (FOD) is a prerequisite for apron- and runway-level safety control. Visible-light monitoring degrades sharply under low illumination, dust, and fog, whereas infrared sensing locks onto heat-emitting targets but lacks scene texture; infrared–visible fusion has therefore become a mainstream route to robust detection [1]. YOLO-family detectors have accordingly been widely adopted for airport apron perception, including small-scale FOD detection [2] and abnormal-behavior recognition under complex apron backgrounds [3,4], and unmanned aerial vehicle imagery has been used for runway inspection and airfield condition assessment [5].

Feature extraction from fused images nonetheless faces three coupled difficulties. Thermal saliency and visible texture remain deeply entangled in the fused feature space, and conventional convolutions lack the selectivity to separate them, so bright backgrounds mask infrared targets or infrared diffusion blurs visible contours; cross-attention approaches such as CrossFuse address this mainly at the fusion stage rather than inside the detector [6]. Modality interference is also scene-dependent and hierarchical, so naively concatenating or summing RGB and infrared features can amplify noise from the weaker modality, calling for layer-wise rather than static recalibration [7]. Finally, the strided-convolution or pooling operations used for downsampling are inherently lossy; recent small/occluded-object detectors have replaced them with space-to-depth-style, information-preserving downsampling paired with an additional high-resolution P2 branch [8]. These three issues jointly explain why a generic YOLO baseline underperforms on fused infrared–visible imagery.

Existing multimodal detection research mainly follows two technical routes: explicitly fusing infrared and visible modalities into a single image before detection, for example, LUT-Fuse distills a fusion network into lightweight, learnable look-up tables for fast, detector-friendly fusion [9], or performing deep cross-modal interaction directly inside the detection backbone without an intermediate fusion image, as in Fusion-Mamba’s state-space modeling of infrared–visible feature interaction [10].

Within the backbone-interaction route, the design space has diversified quickly: iterative cross-attention progressively refines multispectral features [11], cascade alignment-guided transformers correct geometric misregistration before fusion [12], loosely coupled DETR-style architectures balance modality contributions via modality-aware queries [13], and specificity-guided reconstruction separates modality-shared from modality-specific components before recombination [14]. Dual-feature-enhancement designs such as DEYOLO further introduce bi-directional decoupled focus and cross-modality enhancement for RGB-infrared detection [15], and recent one-stage detectors such as YOLO-Adaptor target non-aligned visible–infrared detection with fast adaptive feature alignment [16]. These methods, however, do not explicitly target the extreme scale variation and information loss that arise for distant, low-pixel-count targets. A related body of work addresses small-target loss during downsampling through space-to-depth or channel-transfer operators paired with a shallow detection branch, as in FFCA-YOLO [17], SOD-YOLO [18], LR-Net [19], and MLSA-YOLO [20], with SPD-Conv [21] providing a general space-to-depth building block for low-resolution images and small objects; together these establish an information-preserving, P2-level path as a near-standard component for small-object-sensitive detectors, motivating the Bi-directional Focus module and P2 branch adopted here. Channel and spatial attention modules such as scSE [22] and CBAM [23] provide generic feature reweighting, but they do not explicitly separate thermal-radiation cues from texture-detail cues. A third line targets dynamic rather than static feature recalibration via coordinate/involution attention in the SPPF stage [24], multi-scale attention with feature-pyramid fusion [25], and hierarchical gated multi-scale attention [26]. Static concatenation of heterogeneous features is therefore increasingly insufficient, motivating the explicit, learnable, physically separated recalibration realized by the TTDR module.

Real-time digital twins, BIM-enabled structural monitoring, vision–language frameworks, and retrieval-augmented generation systems have been explored for airport safety information integration [27,28,29,30]; most such systems, however, assume single-modality visible imagery or treat detection accuracy as secondary to integration, which motivates improving the perception front end that feeds them.

In airport airside scenes, small targets such as distant personnel, ground vehicles, and small hazards occupy only a few pixels and have weak contrast against complex backgrounds; missed detections therefore carry direct safety risks. At the same time, an active airport streams dozens of apron and runway cameras through constrained edge hardware, making detection accuracy and computational cost a pair of objectives that must be satisfied jointly.

To address these issues, this paper proposes a thermal-texture decoupling and recalibration object detection algorithm for fused images (DTF-YOLO), built on a thoroughly restructured YOLOv11 backbone. The main contributions are as follows:

1. A Thermal-Texture Decoupling Recalibration (TTDR) module is proposed: parallel decoupled paths separately perceive low-frequency thermal radiation and high-frequency texture detail, dynamically calibrating the weights of infrared and visible features to resolve feature contamination and semantic ambiguity.
2. A fully information-preserving Bi-directional Focus sampling strategy is introduced: a spatial-channel slicing mechanism replaces conventional lossy downsampling, fully preserving the low-level thermal response information.
3. A four-scale feature fusion architecture is constructed: an additional P2, extremely-high-resolution feature layer enhances the model’s ability to detect tiny targets, meeting the practical demands of complex airside and apron scenes.

These components are designed for the scale, contrast, and modality-interference characteristics of airport airside targets. Because no large-scale public airport infrared–visible benchmark with dense small targets is currently available, the method is evaluated on M3FD and LLVIP, which share the relevant small-object and cross-modality challenges; airport-specific end-to-end validation is left for future work.

## 2. Materials and Methods

**Figure 1:**
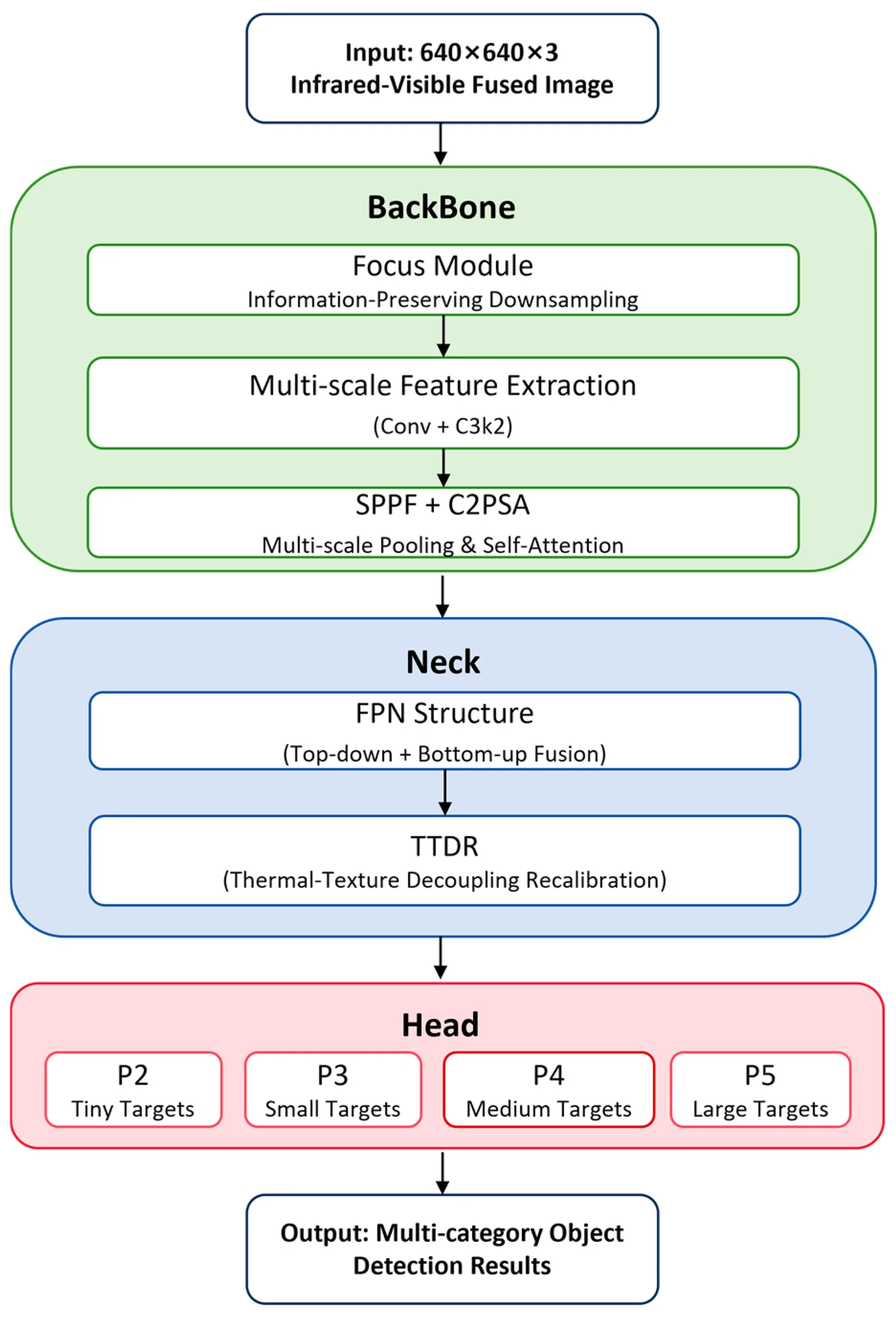
Overall architecture of DTF-YOLO.

As shown in Figure 1, the proposed DTF-YOLO architecture uses YOLOv11 as its baseline backbone and, through modular improvements tailored to the characteristics of fused images, builds a hierarchical detection system comprising feature extraction, cross-modal recalibration, and multi-scale fusion. The overall architecture consists of three core components: the backbone network, the feature-fusion neck, and the multi-scale detection head. The structured output of the detection head (category, confidence, and bounding-box coordinates) provides the basis for downstream inspection and safety analysis.

### 2.1. Overall Network Architecture

At the initial stage of feature extraction, a Bi-directional Focus sampling module is introduced as the first layer of the network; it replaces conventional convolutional downsampling with a spatial slice-transfer mechanism, maximizing the preservation of pixel-level gradient information in the fused image at the very stage where the receptive field is first enlarged. The backbone employs a series of improved C3k2 blocks and C2PSA spatial self-attention modules, which extract deep features from the fused image layer by layer through residual connections and multi-branch convolutional kernels, injecting spatial-weight awareness into the deep semantics so that the network gains stronger discriminative power when processing salient infrared thermal targets against complex visible-light backgrounds.

A Thermal-Texture Decoupling Recalibration module (TTDR) is embedded at the cross-scale connection nodes of the neck. Rather than the conventional direct feature concatenation, this architecture uses the TTDR module to dynamically recalibrate the current-scale features during upsampling and feature alignment. TTDR performs a non-linear decomposition of the fused features via parallel thermal-radiation-perception and texture-detail-perception paths, and dynamically calibrates the contribution weights of infrared saliency signals and visible-light edge gradients according to the semantic needs of the current scale. Through this decoupled recalibration path, the network effectively suppresses redundant background in the fused image while strengthening the representation quality of targets in complex environments.

This paper adopts a multi-scale detection-head architecture specifically optimized for fused scenes. Based on the outputs of the feature pyramid network and the path-aggregation network, the algorithm builds four prediction branches spanning scales P2 to P5, corresponding respectively to detection needs ranging from tiny to large-scale targets. Each prediction branch adopts a decoupled-head design that logically separates the classification task from the bounding-box regression task, reducing the mutual interference between heterogeneous features during multi-task learning. At the loss-computation level, the algorithm integrates Complete-IoU loss with distribution focal loss, together with a training strategy optimized for the color space of fused images, to achieve simultaneous improvement in localization accuracy and semantic discrimination.

### 2.2. Fully Information-Preserving Bi-Directional Focus Module

In wide-field-of-view airside surveillance, a weak thermal-source signal may occupy only a few pixels. Conventional strided-convolution downsampling directly discards a large amount of spatial information, causing severe attenuation of target features early in the backbone. This paper introduces a Bi-directional Focus module that replaces conventional strided convolution with a spatial-channel slice-transfer mechanism. The module decouples the fused image into four sub-images along the horizontal and vertical dimensions, concatenates them along the channel dimension, and then performs cross-channel non-linear fusion via a 1 × 1 convolution. With only a marginal increase in computational cost, this strategy expands the receptive field while preserving low-level pixel gradients, effectively reducing the loss of tiny-target features under such wide-field conditions.

Image downsampling is fundamentally a process of signal compression and feature filtering. In the initial layer of a general-purpose detector, a convolution kernel with stride 2 is typically used, meaning that during the sliding-window process only about 25% of the pixel region is directly centered on by the kernel, while the remaining pixel signals are averaged into the background or discarded outright. For infrared–visible fused images, this approach has two major limitations. First, low-frequency energy loss of salient thermal-infrared targets: the bright response of a small-scale target in the infrared modality may consist of only a few core pixels, and once the sampling point deviates, the target signal can be severely attenuated or even disappear in the first layer of the backbone. Second, aliasing of visible-light edges: the fine texture in a fused image depends mainly on the gradient differences between neighboring pixels, and conventional strided convolution interrupts this continuity, producing jagged edges or artifacts in the reconstructed feature map. This irreversible loss of information entropy greatly limits the detector’s ability to discriminate weak, small, and multiple targets against complex backgrounds.

**Figure 2:**
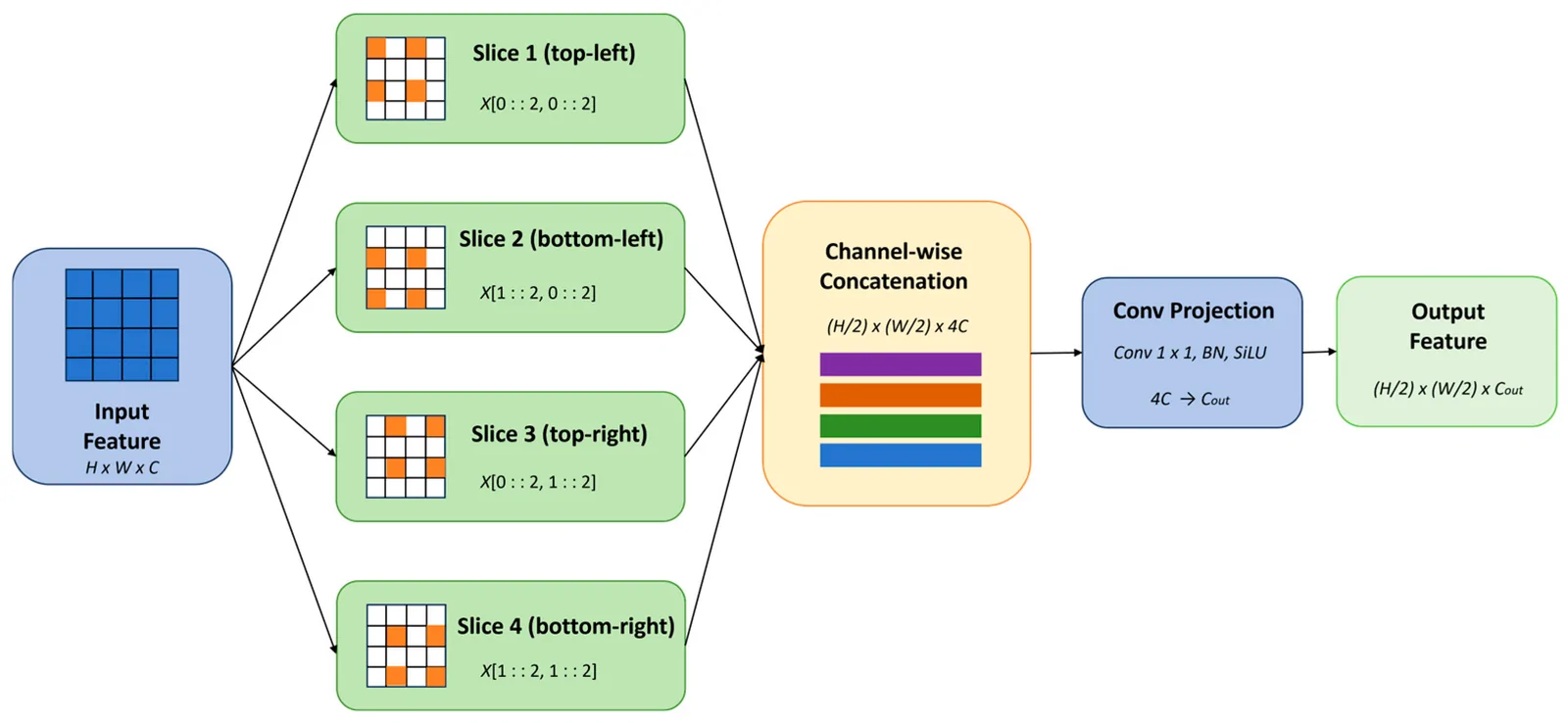
Structure of the Bi-directional Focus module.

As shown in Figure 2, the core logic of the Bi-directional Focus module is to convert spatial redundancy rather than discard spatial information. The module uses a sampling operator with a stride of 2 to decouple the original fused image separately along the horizontal and vertical dimensions, an operation similar to splitting every pixel block of the image into four independent sub-sampled maps. Let the input fused-image tensor be X; the mathematical mapping of its slicing process is as follows: path 1 (top-left sampling) extracts all pixels at even rows and even columns; path 2 (bottom-left sampling) extracts all pixels at odd rows and even columns; path 3 (top-right sampling) extracts all pixels at even rows and odd columns; and path 4 (bottom-right sampling) extracts all pixels at odd rows and odd columns. After this operation, neighboring pixels that were originally spatially adjacent are halved along both the vertical and horizontal dimensions, but their feature information is fully transferred to the channel dimension; the module then concatenates the four branches to generate a reorganized feature tensor. The slicing step itself is a lossless, reversible rearrangement of pixels into the channel dimension, so no raw pixel information is discarded at this stage; the subsequent 1 × 1 convolution then performs a learnable, non-linear fusion of these channels. The term “information-preserving” therefore applies specifically to the slice-rearrangement operation, not to the entire module; the 1 × 1 convolution is a trainable transformation that inevitably mixes and compresses the rearranged channels.

### 2.3. Thermal-Texture Decoupling Recalibration Module

**Figure 3:**
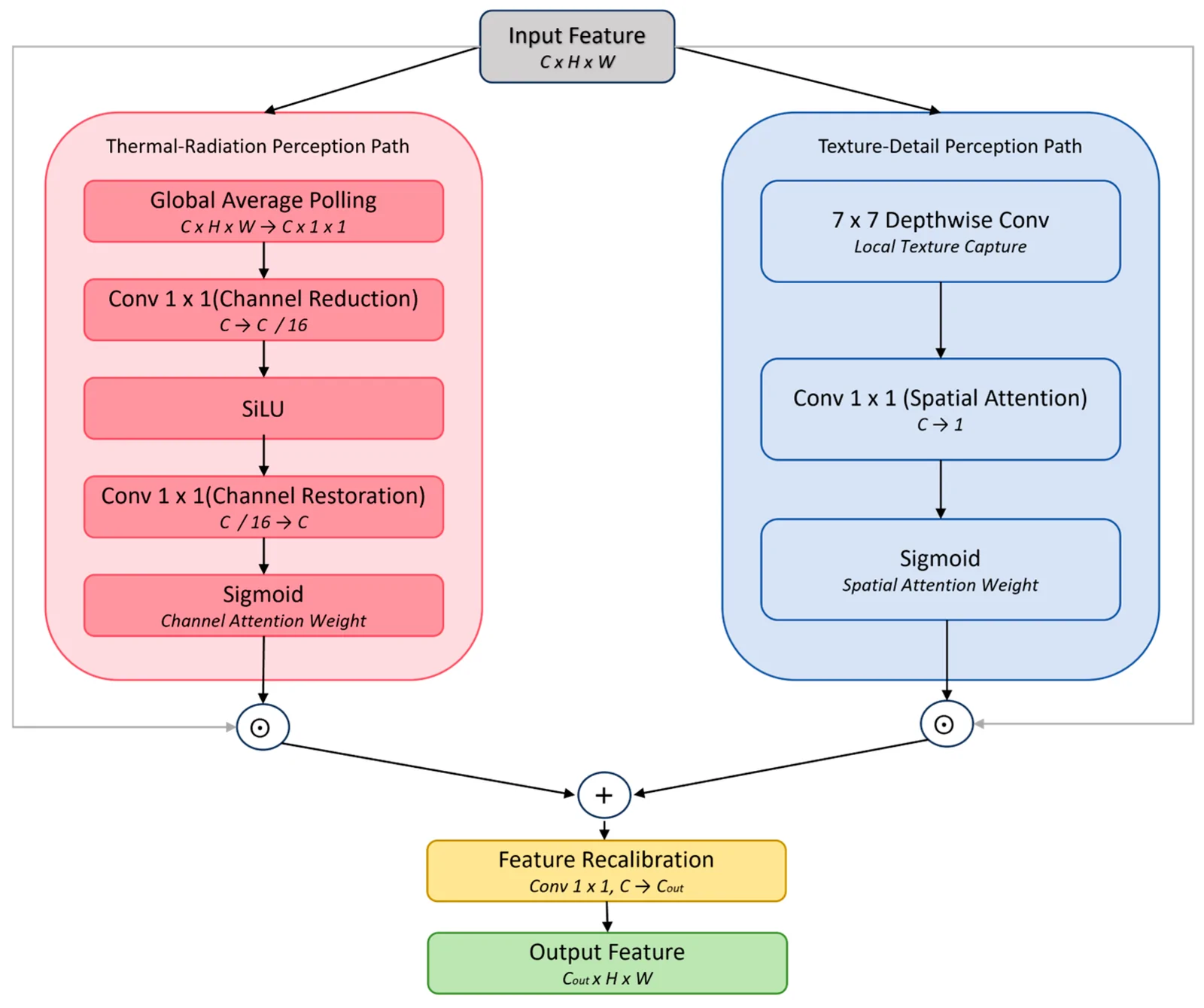
Structure of the Thermal-Texture Decoupling Recalibration (TTDR) module.

In the deep features of a fused image, the low-frequency energy of the infrared modality and the high-frequency detail of the visible modality can easily mask each other, so that, for example, a person’s thermal radiation is interfered with by the background gradient of sun-heated pavement and idling ground-support equipment. To resolve this conflict in deep representation, a Thermal-Texture Decoupling Recalibration module (TTDR) is introduced in the feature-fusion neck, as shown in Figure 3. The module builds two parallel perception paths.

The thermal-radiation-perception path captures the low-frequency saliency information in the image, that is, the energy-concentrated regions of thermal targets. To obtain a global view of the thermal-distribution characteristics, this branch first uses global average pooling to compress spatial information into a corresponding channel descriptor, then uses two successive convolutional layers to build a channel-interaction network that learns non-linear dependencies between channels. This process can be written as

(1) $$W_{thermal}=\sigma ({Conv}_{1\times 1}(SiLU({Conv}_{1\times 1}(GAP(F)))))$$

where $F\in R^{H\times W\times C}$ is the input fused feature, σ(⋅) denotes the sigmoid function, and $W_{thermal}\in R^{1\times 1\times C}$ is a channel-wise weight vector. The weight matrix output by this path is, in essence, a channel descriptor that dynamically boosts the response of channels carrying salient infrared thermal signals, ensuring that the energy features of thermal targets are not gradually weakened or lost as they propagate through the deep network.

The texture-detail-perception path focuses more on capturing local, high-frequency geometric structure. To better extract fine texture information, this branch introduces large-kernel depthwise separable convolution; compared with standard convolution, depthwise separable convolution reduces the parameter count while extracting spatial features independently for each channel, avoiding feature aliasing between different modalities. The use of a large convolution kernel markedly enlarges the local receptive field, enabling the model to perceive high-frequency structures with long-range pixel continuity, such as marking lines and edge contours. This path can be written as

(2) $$W_{texture}=\sigma \left({Conv}_{1\times 1}\left({DWConv}_{7\times 7}(F)\right)\right)$$

where $W_{texture}\in R^{H\times W\times 1}$ is a spatial weight map. The resulting spatial weight map provides pixel-level response enhancement in texture-rich regions of the image.

After obtaining the two sets of weight maps, the TTDR module reweights the input feature independently along each path via element-wise multiplication, producing two reweighted feature maps $F⊙W_{thermal}$ and $F⊙W_{texture}$. The combined output is

(3) $$F_{out}={Conv}_{1\times 1}\left(F⊙W_{thermal}+F⊙W_{texture}\right)$$

where ⊙ denotes broadcast element-wise multiplication. These two reweighted feature maps emphasize complementary physical cues: the thermal path yields a high response for pixel regions dominated by thermal-target energy, while the texture path yields a high response for regions dominated by background or edge detail. These two complementary, reweighted feature maps are then combined by element-wise addition. Because each map has already been selectively reweighted before this step, the addition recombines disentangled, complementary responses rather than allowing raw thermal and texture signals to interfere with one another, as would occur under conventional, unweighted concatenation or summation of the two modalities. This decouple-before-combine mechanism forces the model to first attribute the physical origin of a feature within a single channel and only then integrate it; the final 1 × 1 projection convolution performs deep integration of the combined stream and achieves dynamic normalization of the feature space.

The two paths differ from generic channel or spatial attention modules such as scSE and CBAM. A generic channel-attention module reweights all channels with a single shared excitation mechanism, and a generic spatial-attention module reweights all positions with a single shared spatial map; both are task-agnostic and do not assign different physical roles to the two branches. In TTDR, the channel path and the spatial path receive the same input F but apply fundamentally different operations: the channel path uses global average pooling followed by two 1 × 1 convolutions to capture low-frequency thermal-energy statistics, whereas the spatial path uses a 7 × 7 depthwise separable convolution to capture high-frequency texture continuity. The two paths therefore produce complementary weight descriptors—a channel vector $W_{thermal}\in R^{1\times 1\times C}$ and a spatial map $W_{texture}\in R^{H\times W\times 1}$—that are explicitly tied to two different physical cues. This physical separation, rather than a generic channel-then-spatial attention stack, is what the TTDR module contributes.

### 2.4. Four-Scale Feature Fusion Neck

Targets in this scenario span an extremely wide range of scales, from nearby large aircraft or ground-support vehicles to distant, low-pixel-count personnel and small hazards. In general-purpose detection architectures, the feature pyramid formed by layers P3 (1/8 downsampling), P4 (1/16 downsampling), and P5 (1/32 downsampling) is the mainstream configuration; however, in infrared–visible fused images, the discriminative evidence for some key targets is often hidden in extremely fine pixel-level contrast. For example, in a long-range monitoring scenario, a thermal hotspot indicating a safety hazard may occupy fewer than 4 × 4 pixels in the input image, and by the time the feature reaches the P3 layer after three downsampling stages, the receptive field has expanded so much that the structural features of such tiny targets are heavily diluted by the surrounding texture.

To bridge this gap in spatial representation, this algorithm adds an extra P2 feature branch (1/4 downsampling scale) to the neck. The P2 layer retains a denser spatial arrangement of pixels and can pass the low-level, high-fidelity features captured by the Bi-directional Focus module directly to the detection head. This four-scale layout builds a progressive semantic space from P2 to P5, ensuring that the network can both capture the macro view of a scene and resolve tiny anomalies. This ability to localize tiny targets directly supports reliable small-object detection in complex scenes.

To achieve organic integration of features at different scales, the optimized neck adopts an enhanced bi-directional feature-pyramid path composed of a top-down feature-enhancement path and a bottom-up path-aggregation network. After upsampling, high-level features inject abstract category-level semantics into the low-level feature maps, guiding the low-level network to recognize which high-frequency textures belong to targets of interest and thereby suppressing background noise; low-level features, in turn, feed precise boundary-localization information back to the high level through convolutional downsampling. In the context of fused images, the precise gradient signal provided by the P2 layer can significantly correct localization offsets that arise in high-level semantics.

## 3. Results

### 3.1. Experimental Setup

This paper selects two highly complementary benchmark datasets from the field, M3FD and LLVIP. M3FD contains 4200 infrared–visible image pairs captured under daytime, nighttime, and smoke-occluded conditions, with annotations for six categories: People, Car, Bus, Truck, Lamp, and Motorcycle. LLVIP contains more than 15,000 high-definition infrared–visible image pairs covering streets, parks, parking lots, and building entrances, with fine-grained annotation for the person category. A fixed random split is generated with a ratio of 8:1:1 for M3FD and 6:2:2 for LLVIP, and the same split files are applied to all compared methods. These two datasets are used as proxy benchmarks for infrared-visible small-object detection.

To generate the three-channel fused input, each infrared–visible pair is spatially aligned using the registration provided by the dataset, and both modalities are normalized to [0, 1]. The fused image is constructed by the MS-DAFNet fusion network [31], which produces a three-channel image that preserves the thermal radiation of the infrared modality and the texture detail of the visible modality. The same fusion procedure is applied to all image pairs before training and testing, and no dataset-specific fusion parameter is tuned.

The software environment for the experiments in this study is built on the PyTorch 2.1.0 deep-learning framework, and the hardware platform uses an NVIDIA RTX 4090 GPU with 24 GB of memory. The YOLOv11 baseline is the official YOLOv11n implementation [32], and all models are trained from the same pretrained COCO weights under identical settings. Stochastic gradient descent is used as the optimizer, with an initial learning rate of 0.01 and a momentum factor of 0.937, and a cosine-annealing schedule dynamically adjusts the learning rate over 150 training epochs. The batch size is set to 16, the input resolution is 640 × 640, and the main experiments use a fixed random seed of 42. To ensure a rigorous statistical evaluation, all compared methods are trained and evaluated three times with different random seeds (42, 123, and 2026), and the mean and standard deviation across the three seeds are reported. To address the overfitting risk introduced by the coupling of thermal-feature saliency and background texture in fused images, the weight-decay coefficient is raised from the common value of 0.0005 to 0.001; this stronger regularization constraint guides the network to mine the intrinsic physical properties of targets rather than capturing spurious correlations in the synthetic data. A label-smoothing factor of 0.1 is also introduced. For data augmentation, HSV perturbation is applied with small hue, saturation, and value factors to preserve the physical consistency between thermal radiation and spatial gradients in the fused images. Mosaic is enabled with probability 1.0 and Mixup with probability 0.1 during the main training phase, and Mosaic is disabled during the final 20 epochs to allow the model to perform pixel-level boundary regression on clean feature spaces. During evaluation, the confidence threshold is set to 0.001 for mAP computation, and NMS is performed with an IoU threshold of 0.45.

### 3.2. Results on the M3FD Dataset

**Figure 4:**
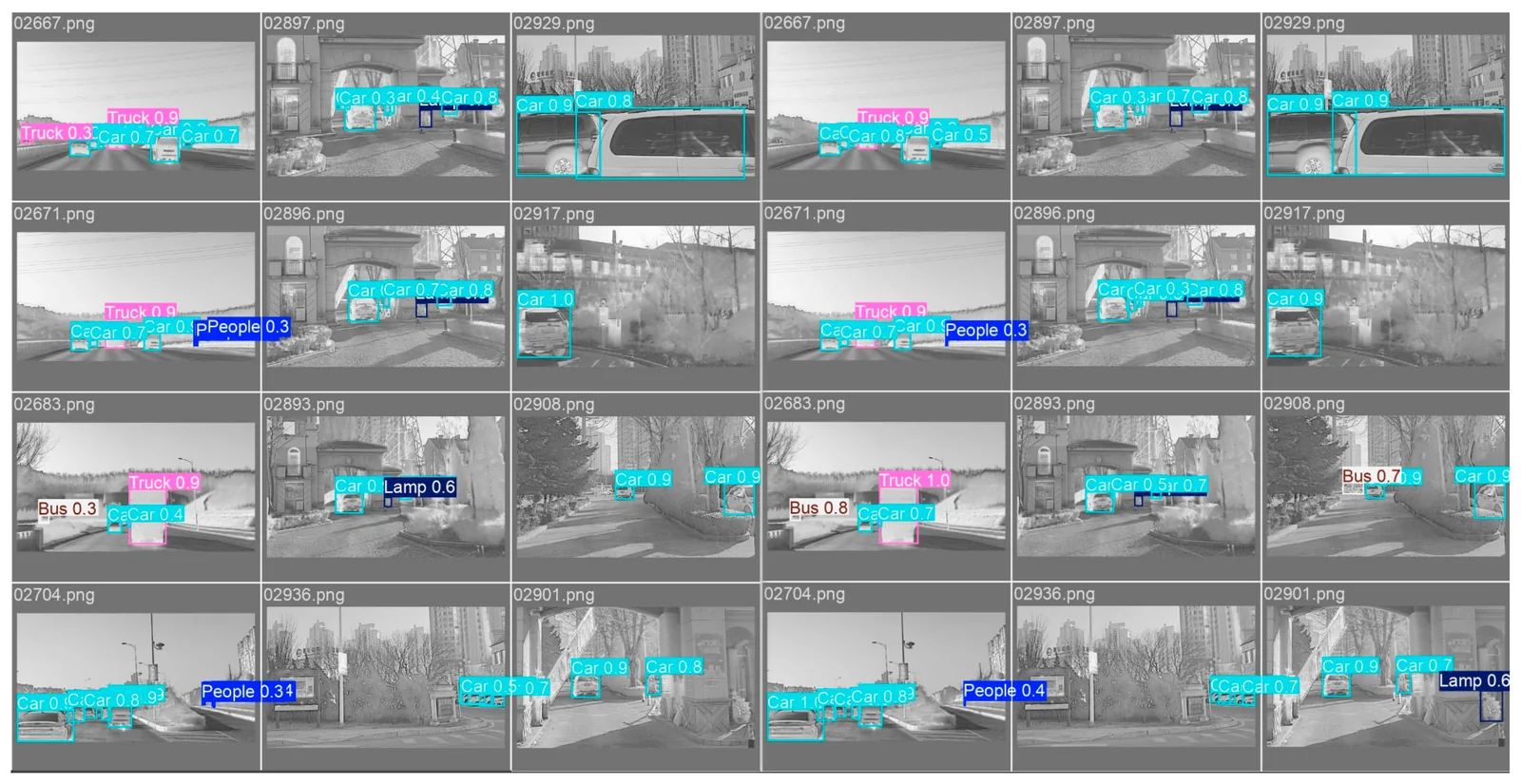
Sample detection results of the proposed method on M3FD.

Table 1 and Figure 4 show the comparative results on M3FD. GFLOPs and FPS were measured on the same RTX 4090 GPU under PyTorch (FP32) with an inference batch size of 1 and a 640 × 640 input, as computational-efficiency indicators for the edge-oriented design. Relative to the two-stage Faster-RCNN detector and the classic single-stage YOLOv8 model, the proposed algorithm shows a dual advantage of high accuracy and light weight. In terms of detection performance, its mAP@0.5 leads Faster-RCNN (51.11%) and YOLOv8 (80.70%) by 36.21 and 6.62 percentage points, respectively, and precision also reaches the best value of 89.54%. In terms of model complexity, Faster-RCNN and YOLOv8 have parameter counts as high as 64.39 M and 11.10 M, respectively, with GFLOPs of 17 and 8.6 and FPS of 36 and 103; the proposed algorithm achieves higher accuracy with a far smaller parameter budget and lower computational cost. Furthermore, against the dedicated heterogeneous-feature-fusion detection framework YOLOFuse, the proposed algorithm leads by 4.87 percentage points in mAP@0.5 while using only about 56% of its parameters, reducing GFLOPs from 8.2 to 6.3 and increasing FPS from 92 to 117, which indicates that the proposed algorithm achieves more effective cross-modal representation with greater computational efficiency when handling complex heterogeneous data. Compared with the YOLOv11 baseline, the overall mAP@0.5 rises from 82.01% to 87.32%, a gain of 5.31 percentage points, at the cost of only a marginal increase in GFLOPs (from 6.1 to 6.3) and a slight decrease in FPS (from 121 to 117). On the stricter localization metric mAP@0.5:0.95, the algorithm also improves from 56.61% to 57.78%, confirming the effectiveness of the P2-P5 four-scale feature fusion structure in improving bounding-box fit. Meanwhile, recall rises from 73.82% to 80.33%, indicating that by introducing the thermal-texture decoupling recalibration module, the proposed algorithm successfully captures a large number of weak-contrast targets that the baseline model missed owing to feature masking. Stably capturing such targets is important for reliable small-object detection in complex scenes. Although the parameter count grows from 2.58 M to 2.91 M owing to the additional cross-modal perception introduced, this represents, overall, a small increase in computational cost in exchange for a marked improvement in detection accuracy.

Table 2 further reports the per-class AP on M3FD. Faster-RCNN achieves only 9.94% AP on the Lamp category, whereas the proposed method raises it to 48.62%; this indicates that a conventional CNN without deep cross-modal fusion struggles with tiny targets whose infrared and visible appearances differ greatly. Compared with the dedicated fusion detector YOLOFuse, the proposed method still shows clear advantages on several key categories. DTF-YOLO improves the People AP by 2.08 percentage points over YOLOv11 and also outperforms YOLOFuse on this category. On Motorcycle, it gains 5.39 and 2.81 percentage points over YOLOv11 and YOLOv8, respectively. The largest improvement appears on Lamp, where the AP is 6.59 and 3.77 percentage points higher than YOLOv11 and YOLOFuse, respectively. Lamp targets usually appear as tiny geometric points in the visible modality and as strong localized radiation in the infrared modality; the texture path of TTDR enhances their edge gradients, while the thermal path locks onto their radiation cores, which alleviates the insufficient response of conventional convolution to heterogeneous feature entanglement. For large, texture-simple categories such as Bus, the accuracy fluctuates slightly, indicating that the decoupling mechanism, while strengthening tiny heterogeneous features, has a mild suppression effect on overly homogeneous large structures; this balance remains favorable for the overall detection task.

It should be noted that recent multimodal detectors such as DEYOLO [15] and YOLO-Adaptor [16] are discussed in the Introduction, but they are not included in Table 1. This is because their official implementations were not available for a controlled comparison under identical training and inference settings; a structural comparison with these methods is instead provided in Section 4.

### 3.3. Results on the LLVIP Dataset

**Figure 5:**
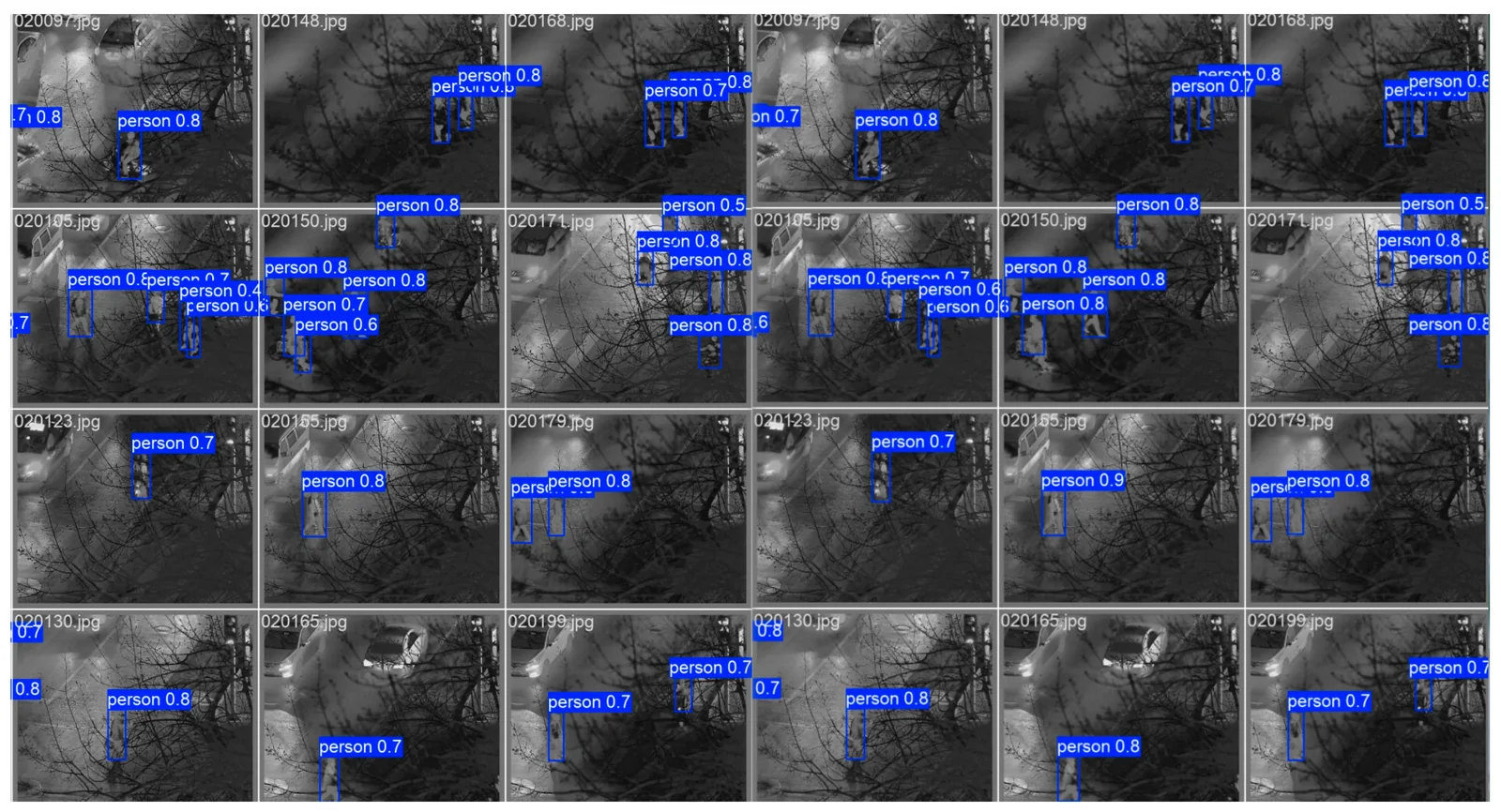
Sample detection results of the proposed method on LLVIP.

As shown in Table 3 and Figure 5, the proposed method achieves a performance improvement on LLVIP that is consistent with that on M3FD, but the margin of improvement is smaller. This can be attributed to three objective factors: first, the YOLOv11 baseline already reaches a high mAP@0.5 of 94.84% on this dataset, leaving relatively limited room for further gains; second, M3FD involves a more complex multi-class detection task with six categories, where the thermal-texture decoupling mechanism can play a larger role; third, M3FD contains a large number of small targets, whereas LLVIP focuses mainly on medium-scale pedestrian targets, so the improvement in small-target detection brought by the proposed method is more evident on M3FD.

Table 3 also reports the computational efficiency on LLVIP. DTF-YOLO maintains the same GFLOPs and FPS as reported on M3FD (6.3 GFLOPs and 117 FPS), and compared with YOLOv11 (6.1 GFLOPs, 121 FPS), it achieves a 0.42 percentage point gain in mAP@0.5 at a marginal computational cost.

Figure 5 shows the detection results of the baseline model (left three columns) and DTF-YOLO (right three columns) in typical nighttime scenes of LLVIP. The proposed method successfully detects all visible pedestrians with higher confidence scores, whereas the baseline model detects the main pedestrian targets but with relatively lower confidence. The visualization further confirms the stability of DTF-YOLO on LLVIP. Although the absolute gain is smaller than that on M3FD, the method consistently improves over a strong baseline, indicating its robustness across different infrared–visible datasets.

### 3.4. Ablation Study

To verify the contribution of each proposed module to detection performance on infrared–visible fused images, a systematic ablation study was conducted. The effectiveness of Bi-directional Focus sampling (Focus), the Thermal-Texture Decoupling Recalibration module (TTDR) at different levels, and the four-scale detection architecture (4-Scale) are analyzed in detail.

The results are shown in Table 4. Comparing the baseline model with the variant that adds only the Focus module (+Focus), mAP@0.5 rises from 82.01% to 83.45% and recall increases by 1.38 percentage points, confirming that replacing conventional convolutional downsampling with the spatial-channel slice-transfer mechanism more effectively locks in low-level pixel-wise gradients. In the level-wise ablation of the TTDR module, inserting the recalibration mechanism alone at either P3 or P4 brings a clear performance gain, with +TTDR (P4) (84.65%) slightly outperforming +TTDR (P3) (84.28%), which suggests that deep semantic features have a more urgent need for cross-modal decoupling. When TTDR is applied simultaneously at both P3 and P4 (TTDR P3 + P4), mAP@0.5 rises to 85.92% and recall reaches 78.95%. Further inserting TTDR at the P5 level (TTDR P3 + P4 + P5) yields a marginal change in mAP@0.5 to 85.88%, indicating that the deepest semantic layer already relies less on cross-modal separation and that the P3 + P4 configuration is sufficient.

To isolate the interaction between modules, three additional combinations are evaluated. Applying Focus together with TTDR (Focus + TTDR) achieves 86.45% mAP@0.5, which confirms that preserving low-level gradients provides cleaner inputs for the subsequent decoupling recalibration. Adding the P2 branch to TTDR (TTDR + 4-Scale) reaches 86.52% mAP@0.5, showing that the high-resolution branch and cross-modal recalibration complement each other. When Focus and the 4-Scale structure are combined without TTDR (Focus + 4-Scale), the model reaches 85.10% mAP@0.5, which is lower than the TTDR-inclusive variants, indicating that expanding the scale range alone is insufficient without effective modality decoupling.

When all of the proposed innovations are combined (Ours), the model reaches its best performance, with mAP@0.5 stabilizing at 87.32%, a gain of 5.31 percentage points over the baseline, and recall rising to 80.33%, a gain of 6.51 percentage points over the baseline.

It is worth noting that the full model’s mAP@0.5:0.95 is 57.78%, which is slightly lower than the 58.15% achieved by +TTDR (P3 + P4). This does not indicate a degradation in overall detection capability, but rather a trade-off between detection recall and strict localization precision. The P2 branch and the Bi-directional Focus module introduce high-resolution, low-level features that substantially improve the detection of tiny targets (as reflected by the 1.38 percentage point recall gain over the baseline), but these high-resolution features also carry more background noise and boundary ambiguity. Under the strict IoU thresholds (0.5:0.95), a small number of large-object bounding boxes receive slightly looser fits, causing a minor drop in the strict localization metric. However, this sacrifice in marginal bounding-box precision is outweighed by the significant gains in mAP@0.5 and recall, which are more critical for safety inspection tasks where missing a target is more costly than a minor localization offset.

## 4. Discussion

The experimental results support the two hypotheses behind DTF-YOLO’s design. First, decoupling thermal saliency from texture detail resolves feature contamination more efficiently than direct feature concatenation: the model matches or exceeds YOLOv11’s accuracy with only a marginal parameter increase (from 2.583 M to 2.913 M), and clearly outperforms YOLOFuse with 44% fewer parameters. Second, information loss during early downsampling appears to be a major bottleneck for detecting weak thermal sources, as evidenced by the larger accuracy gain on the small-target-heavy M3FD dataset and by the independent recall contributions of the P2 branch and the Bi-directional Focus module. The improved accuracy-to-parameter ratio also matters in practice, since airport cameras often run on constrained edge hardware, where a lighter model with higher recall is easier to deploy at scale.

The structural design of the proposed modules also distinguishes them from existing approaches. Compared to DEYOLO [15], which introduces bi-directional decoupled focus and cross-modality enhancement at the backbone level, the proposed TTDR operates on the fused feature maps at the neck, separating thermal and texture cues through parallel channel and spatial perception paths. Compared to generic downsampling operators such as SPD-Conv [21], the Bi-directional Focus module preserves information via four-directional slicing and channel rearrangement, which differs from SPD-Conv’s space-to-depth followed by non-strided convolution. Furthermore, unlike generic attention modules such as scSE [22] and CBAM [23], which apply task-agnostic channel or spatial reweighting, TTDR explicitly assigns different physical roles to its two branches: the channel path captures low-frequency thermal statistics, while the spatial path captures high-frequency texture continuity.

Despite these contributions, this study has several limitations. First, because no large-scale public airport infrared–visible benchmark with dense small targets is currently available, the method is evaluated on M3FD and LLVIP as proxy benchmarks. Airport-specific end-to-end validation with calibrated camera parameters remains future work. Second, due to computational constraints, a systematic replacement ablation against SPD-Conv, scSE, and CBAM was not conducted; the comparison here is structural rather than numerical. Third, the geospatial mapping pathway sketched in the original manuscript has been removed, as validating its localization accuracy requires calibrated real-world site data that are outside the scope of this study. Future work will focus on collecting a calibrated airport dataset and conducting a broader experimental comparison with recent multimodal detectors to bridge these gaps.

## 5. Conclusions

Motivated by airside safety monitoring, this paper addresses the coupling of thermal saliency and texture detail and the loss of small-target information, during downsampling, in infrared–visible fused images. It also proposes a lightweight detection algorithm, DTF-YOLO. The Bi-directional Focus module preserves low-level thermal-source gradients through lossless spatial-channel slicing, the Thermal-Texture Decoupling Recalibration (TTDR) module dynamically balances heterogeneous modal features via parallel thermal- and texture-perception paths, and the additional P2 branch extends the detection architecture to four scales for improved sensitivity to distant personnel and small hazards. With only 2.91 M parameters, DTF-YOLO achieves 87.32% mAP@0.5 on M3FD and 95.26% on LLVIP, outperforming the YOLOv11 baseline and the dedicated fusion detector YOLOFuse, while adding only a marginal parameter overhead over the baseline and remaining substantially lighter than YOLOFuse. The reported gains are consistent across three independent random seeds.

Building on the limitations identified above, future work will prioritize two directions. First, we will collect a calibrated, site-collected airport infrared–visible dataset with geo-referenced annotations and broader environmental coverage (e.g., rain, fog, heat-haze distortion), which would allow the airport-specific validity of the detector to be assessed under compound extreme conditions. Second, we will conduct a systematic replacement ablation against Focus/SPD-Conv, scSE, and CBAM and evaluate the model on embedded edge devices to quantify practical deployment latency and power consumption.

## Figures and Tables

**Table 1:** Average metric values (%) of each method on the M3FD dataset.

Method	mAP@0.5	mAP@0.5:0.95	Precision	Recall	Params (M)	GFLOPs	FPS
Faster-RCNN	51.11 ± 0.27	26.60 ± 0.33	50.65	53.62	64.39	17	36
YOLOv8	80.70 ± 0.22	56.45 ± 0.16	86.50	76.80	11.10	8.6	103
YOLOv11	82.01 ± 0.18	56.61 ± 0.22	87.46	73.82	**2.58**	**6.1**	**121**
YOLOFuse	82.45 ± 0.19	56.26 ± 0.21	85.31	78.44	5.20	8.2	92
**DTF-YOLO (ours)**	**87.32** ± 0.17	**57.78** ± 0.16	**89.54**	**80.33**	2.91	6.3	117

**Table 2:** Per-class AP (%) on M3FD.

Class	Faster-RCNN	YOLOv8	YOLOv11	YOLOFuse	DTF-YOLO (Ours)
People	50.02	50.40	48.88	49.25	**50.96**
Car	62.28	66.30	67.28	65.81	**67.31**
Bus	72.76	76.80	**80.23**	77.10	72.57
Motorcycle	46.01	44.90	42.32	43.12	**47.71**
Lamp	9.94	41.60	42.03	44.85	**48.62**
Truck	56.62	58.70	58.92	57.44	**59.62**
mAP@0.5:0.95	49.61	56.45	56.61	56.26	**57.78**

**Table 3:** Average metric values (%) of each method on the LLVIP dataset.

Method	mAP@0.5	mAP@0.5:0.95	Precision	Recall	Params (M)	GFLOPs	FPS
Faster-RCNN	86.42 ± 0.31	45.18 ± 0.26	85.33	82.20	64.39	17	36
YOLOv8	94.50 ± 0.18	58.30 ± 0.15	94.12	84.15	11.10	8.6	103
YOLOv11	94.84 ± 0.15	60.67 ± 0.21	95.51	88.45	**2.58**	**6.1**	**121**
YOLOFuse	94.72 ± 0.24	60.15 ± 0.17	94.92	88.16	5.20	8.2	92
**DTF-YOLO (ours)**	**95.26** ± 0.13	**61.58** ± 0.15	**95.86**	**88.91**	2.91	6.3	117

**Table 4:** Ablation study results (%).

Experiment	Focus	TTDR (P3)	TTDR (P4)	TTDR (P5)	4-Scale	mAP@0.5	mAP@0.5:0.95	Recall
Baseline	×	×	×	×	×	82.01	56.61	73.82
+Focus	√	×	×	×	×	83.45	57.12	75.20
+TTDR (P3)	×	√	×	×	×	84.28	57.45	76.85
+TTDR (P4)	×	×	√	×	×	84.65	57.68	77.30
+TTDR (P3 + P4)	×	√	√	×	×	85.92	58.15	78.95
+TTDR (P3 + P4 + P5)	×	√	√	√	×	85.88	58.22	78.80
+4-Scale	×	×	×	×	√	83.78	57.35	76.10
+Focus + TTDR	√	√	√	×	×	86.45	58.02	79.50
+TTDR + 4-Scale	×	√	√	×	√	86.52	58.25	79.85
+Focus + 4-Scale	√	×	×	×	√	85.10	57.60	77.40
**DTF-YOLO (ours)**	**√**	**√**	**√**	×	**√**	**87.32**	**57.78**	**80.33**

## Data Availability

The M3FD and LLVIP datasets used in this study are publicly available benchmark datasets. They can be accessed through their original publications at https://doi.org/10.1109/CVPR52688.2022.01380 and https://doi.org/10.1109/ICCV48922.2021.00160, respectively.

## Abbreviations

The following abbreviations are used in this manuscript:

YOLO	You Only Look Once
TTDR	Thermal-Texture Decoupling Recalibration
CIoU	Complete Intersection over Union
DFL	Distribution Focal Loss
IoU	Intersection over Union
mAP	mean Average Precision
SGD	Stochastic Gradient Descent
